# The role of idealisations in describing an isolated molecule

**DOI:** 10.1007/s10698-019-09342-7

**Published:** 2019-09-24

**Authors:** Vanessa A. Seifert

**Affiliations:** University of Bristol, Bristol, UK

**Keywords:** Idealisations, Relation between chemistry and quantum mechanics, Stability, Molecular structure

## Abstract

The investigation of the relation between chemistry and quantum mechanics includes examining how the two theories each describe an isolated molecule. This paper focuses on one particular characteristic of chemistry’s and quantum mechanics’ descriptions of an isolated molecule; namely on the assumptions made by each description that an isolated molecule is stable and has structure. The paper argues that these assumptions are an idealisation. First, this is because stability and structure are partially determined by factors that concern the context in which a molecule is considered (i.e. thermodynamic conditions, time-range of experiment, environment, etc.). Secondly, the stability and structure of a molecule can only be empirically identified with reference to those factors. This paper examines these assumptions in the context of the philosophical literature on idealisations. This examination is a novel contribution that raises interesting questions about the relation between the two theories, the nature of stability and structure, and the function of these assumptions in the two theories.

## Introduction

When chemistry and quantum mechanics each describe an isolated molecule, they assume that the molecule is stable and has structure. Identifying these assumptions as an idealisation is a novel contribution to the discussion of idealisations in chemistry and in quantum mechanics, and raises interesting philosophical issues, including those concerning the relation between chemistry and quantum mechanics.

The paper is structured as follows. “The philosophical investigation of idealisations” section presents how idealisations are understood and discussed in philosophy. “Stability and structure in chemistry and quantum mechanics” section explains why regarding an isolated molecule as being stable and having structure is an idealisation. When it comes to describing an isolated molecule, both chemistry and quantum mechanics make this idealisation. “Philosophical implications” section presents how this idealisation contributes to the investigation of various philosophical issues. Lastly, “Conclusion” section concludes this paper by restating the paper’s main claim.

## The philosophical investigation of idealisations

Making idealisations is one of the most common practices in science and numerous cases can be invoked as examples. ‘(F)rictionless planes, point masses, infinite velocities, isolated systems, omniscient agents, and markets in perfect equilibrium’ are regarded as idealisations that are made in the relevant theories or models (Frigg and Hartmann 2012, Section 1.1). Assuming that gas molecules are infinitely small, or that light consists of ‘one-dimensional beams rather than waves’ are also examples of idealisations (Strevens 2017, p. 2).

The philosophy of science examines various questions regarding idealisations. For example, it examines the nature of idealisations and whether there are distinct kinds of idealisations in science. Moreover, it examines how particular figures in the history of science have understood idealisations. This is because idealisations are crucial to the work of central figures such as Aristotle, Galileo, and Newton (McMullin 1985, pp. 240–262). For example, McMullin analyses Galileo’s understanding of idealisations in order to identify a particular type of idealisation; namely Galilean idealisation (1985). Furthermore, the use of idealisations in science is often examined in relation to philosophical topics such as scientific realism, emergence, models in science, representation and explanation (Ladyman 2008, pp. 360–365). For example, the use of idealisations in science is used as putative evidence for antirealism (Weisberg 2007, p. 657; Cartwright 1983). On the other hand, it is also argued that idealisations can support scientific realism (Weisberg 2007, p. 657; Norton 2012, p. 211).

Perhaps as is to be expected, there is no standardly accepted single account of idealisations. As Weisberg states:

Philosophers of science increasingly recognize the importance of idealization (…). Yet this recognition has not yielded consensus about the nature of idealization. The literature of the past 30 years contains disparate characterizations and justifications, but little evidence of convergence towards a common position. (2007, p. 639)

The lack of a common position about idealisations becomes apparent from the fact that numerous definitions of idealisations are given. For example, Frigg and Hartmann propose the following definition:

An idealization is a deliberate simplification of something complicated with the objective of making it more tractable. (2012, Section 1.1)

McMullin defines idealisations in a similar manner; namely as ‘a deliberate simplifying of something complicated (a situation, a concept, etc.) with a view to achieving at least at a partial understanding of that thing’ (1985, p. 248). Norton argues that idealisations should be taken to ‘refer to new systems some of whose properties approximate some of those of the target system’ (Norton 2012, p. 207), whereas Weisberg defines idealisations as ‘the intentional introduction of distortion into scientific theories’ (Weisberg 2007, p. 639). These definitions illustrate that idealisations are ‘a feature of both the formulation of laws and theories and of their application to the world’ (Ladyman 2008, p. 358). For example, Weisberg understands idealisations as a feature of scientific theories, whereas Norton understands them as a feature of the system or phenomenon that scientific theories describe.^1^

A question which is raised about idealisations concerns their function in science. According to Frigg and Hartmann, the function of idealisations is to make ‘something’ more ‘tractable’ (2012, Section 1.1). Other philosophers specify in more detail the function of idealisations. For example, idealisations are used in order to:

1. ‘reduce the complexity of the model’, by either ‘making the mathematics more tractable or by reducing the empirical demands of the model’ (Strevens 2017, p. 3);
2. identify the ‘core or primary causal factors that give rise to the phenomenon of interest’ (Weisberg 2007, p. 651); and,
3. construct ‘a single model for a particular target or class of target phenomena’ (Weisberg 2007, p. 655).

Moreover, a particular notion of idealisation is often supported with respect to the description of a particular phenomenon. For example, Batterman proposes the notion of ‘infinite idealizations’ with respect to phase transitions and critical phenomena (2011, p. 1033). Weisberg proposes the notion of ‘multiple-models idealization’ regarding the description of global weather patterns (2007, p. 646). McMullin argues that the application of geometry for the specification of the movement of planets is an example of ‘mathematical idealization’ (1985, pp. 248–254).

There are disagreements about several issues regarding idealisations. For example, some argue that idealisations are distinct from approximations, whereas others use the two terms interchangeably.^2^ Also, there are particular examples of theories, models, or representations for which it is not very clear whether idealisations or some other notion (such as analogues) accurately specifies their ‘representational styles’ (Frigg and Hartmann 2012, Section 1.1).^3^ Another issue that is discussed concerns the categorisation of idealisations. For example, Frigg and Hartmann argue that there are two main kinds of idealisations; i.e. ‘Galilean’ and ‘Aristotelian idealisations’ (2012, Section 1.1). McMullin distinguishes between ‘mathematical’, ‘Galilean’ (which are further distinguished into ‘construct’ and ‘causal idealisations’), ‘material’, ‘formal’ and ‘subjunctive idealisations’ (McMullin 1985; Ladyman 2008, pp. 361–362). Weisberg argues that there are three kinds of idealisations; namely ‘Galilean’, ‘minimalist’, and ‘multiple-models idealizations’ (2007). Strevens examines ‘asymptotic’ and ‘simple idealisations’ (2017). Whether there is a unique classification that underwrites all proposed kinds of idealisations is not examined here. Regardless of the exact characterisation of ‘idealisation’ and its various species and cognates, the way chemistry and quantum mechanics describe the stability and structure of an isolated molecule counts as idealisation of some kind in the sense of all the accounts above.^4^

Lastly, a number of examples from chemistry and quantum mechanics have been invoked when discussing idealisations in science. For example:

1. The use of approximate wavefunctions in order to calculate the properties of molecules, is regarded as an example of Galilean idealisation (Weisberg 2007, p. 641).
2. Treating ‘the vibrating bond as spring-like with a natural vibrational frequency’ in order to calculate the vibrational properties of a covalent bond, is regarded as a minimalist idealisation (Weisberg 2007, p. 644).
3. The description of atoms and molecules via the molecular orbital or the valence bond approach is regarded as an example of Weisberg’s third kind of idealisation; i.e. the multiple-model idealisation (Weisberg 2007, p. 646).
4. The Bohr image of the atom is regarded as an example of Galilean idealisation (McMullin 1985, p. 260).
5. The Lewis electron pair model of chemical bonding is also regarded an idealisation (Weisberg 2007, p. 650).

While this paper does not examine the general role of idealisations in chemistry and in quantum mechanics, the above examples illustrate that both chemistry and quantum mechanics employ various idealisations of possibly different types. Moreover, some of the above examples illustrate that there are particular features of the quantum mechanical description of an isolated molecule which have been examined in terms of the notion of idealisations. Nevertheless, there is a particular feature of quantum mechanics (and of chemistry) that has not been identified as an idealisation in either the philosophy of science or the philosophy of chemistry literature; namely the assumptions that an isolated molecule is stable and has structure.^5^

## Stability and structure in chemistry and quantum mechanics

This section presents how stability and structure are defined, understood, and empirically identified.^6^ This analysis explicates why the assumption of an isolated molecule with stability and structure is an idealisation. Specifically, this assumption is correctly construed as an idealisation for two main reasons. First, stability and structure are partially determined by factors that concern the context in which a molecule is considered (i.e. thermodynamic conditions, time-range of experiment, environment, etc.).^7^ Secondly, the stability and structure of a molecule can only be empirically identified with reference to those factors.

Note that a particular account of idealisations regarding their nature, function, etc. is not currently assumed. The aim of this section is to present the scientific evidence which supports the claim that this assumption is an idealisation.

### Stability

There are two cases in which stability is considered with respect to a single molecule, namely:

- the stability of a molecule when considered as part of an ensemble; and,
- the stability of a molecule in isolation.

IUPAC (2014) does not provide a definition of stability with reference to a molecule (whether isolated or not).^8^ However, it is common practice in chemistry to talk about a molecule’s stability. Regarding the stability of a molecule that is part of an ensemble, a molecule is stable in virtue of being part of a stable chemical species, and not in virtue of being in its ground state.^9^ This is because the molecule that is part of an ensemble is neither static, nor always fixed at its ground state. A molecule that is part of an ensemble interacts both intermolecularly and intramolecularly, and its conformation dynamically changes over time.^10^ Therefore, when one refers to a molecule (which is part of an ensemble) as being stable, what is meant is that the molecule is part of a stable chemical species.

Given this, it follows that the stability of a molecule is partially determined by the factors that determine the stability of the chemical species of which the molecule is part. These factors become apparent from the definition of stability with reference to chemical species:

As applied to chemical species, the term expresses a thermodynamic property, which is quantitatively measured by relative molar standard Gibbs energies. A chemical species A is more stable than its isomer B if Δ_r_G^o^ > 0 for the (real or hypothetical) reaction A ≥ B, under standard conditions. If for the two reactions:

$$P \ge {\text{X}} + {\text{Y}}\left( {\Delta_{{\rm r}} {\text{G}}_{1}^{{\rm o}} } \right)$$

$${\text{Q}} \ge {\text{X}} + {\text{Z}}\left( {\Delta_{{\rm r}} {\text{G}}_{2}^{{\rm o}} } \right)$$

$\Delta_{{\rm r}} {\text{G}}_{1}^{{\rm o}} > \Delta_{{\rm r}} {\text{G}}_{2}^{{\rm o}}$, *P* is more stable relative to the product Y than is Q relative to Z.

Both in qualitative and quantitative usage the term stable is therefore always used in reference to some explicitly stated or implicitly assumed standard. (IUPAC 2014, p. 1432)

Based on the above, the stability of a molecule which is part of a chemical species is relative to the following factors:

- The particular reaction(s) with respect to which the stability of the relevant chemical species is considered. For example, while a chemical species *P* is more stable relative to Y when considered with respect to how stable Q is relative to Z, *P* is less stable relative to Y when considered with respect to how stable A is relative to T (for the hypothetical reaction A ≥ X + T, $\Delta_{{\rm r}} {\text{G}}_{3}^{{\rm o}}$, where $\Delta_{{\rm r}} {\text{G}}_{3}^{{\rm o}} > \Delta_{{\rm r}} {\text{G}}_{1}^{{\rm o}}$).
- The particular chemical species with which the relevant chemical species’ stability is relative. For example, while *P* is more stable relative to Y (when considered with the reaction Q ≥ X + Z), it is less stable relative to M (when considered with the reaction Q ≥ X + Z), if it is the case that there is a hypothetical reaction *P* ≥ X + M (with $\Delta_{{\rm r}} {\text{G}}_{4}^{{\rm o}}$) such that $\Delta_{{\rm r}} {\text{G}}_{4}^{{\rm o}} < \Delta_{{\rm r}} {\text{G}}_{2}^{{\rm o}}$.
- The thermodynamic conditions in which those reactions are performed. The relative molar Gibbs energies are calculated and compared under the assumption that the considered reactions are performed under standard thermodynamic conditions. However, if the thermodynamic conditions are different (for example, the temperature or pressure of the examined systems are different), then it is possible that the relevant molar Gibbs energies are different, and thus that the stability of the chemical species is different.^11^
- The environment in which that particular set of reaction(s) is assumed to be performed. This includes the specification of the type of solution or background gas within which the reactions are performed. For example, there are polymers which ‘are reported to be stable in air up to 500 °C and to nearly 800 °C in nitrogen’ (Marvel 2009, p. 361).

Given the above, the stability of a molecule that is part of an ensemble is a relational property of that molecule; namely a property which the molecule has ‘in relation to other things’ (Ney 2014, p. 285).^12^ This is because the molecule has this property in virtue of the relation that the relevant chemical species has with other chemical species, as well as in relation to particular thermodynamic conditions, to a set of reaction(s), and to the environment in which the relevant chemical species is assumed to be found.

The other case considered here is a molecule in isolation. Contrary to a molecule which is part of an ensemble, one would expect the stability of an isolated molecule to be an intrinsic property which is only determined by its composing entities and interactions.^13^ In this case, the stability of a molecule in isolation would refer to the ground state of the molecule; namely to ‘the state of lowest Gibbs energy’ (IUPAC 2014, p. 646). This is because the ground state of a molecule is taken to correspond to a particular internal structure of the molecule. This structure corresponds to the average nucleonic and electronic configurations of the atomic nuclei and electrons within the molecule, and is determined by their respective interactions. However, the in principle impossibility of empirically identifying any property of an isolated system, entails that this model of the stability of an isolated molecule (specified solely in terms of the molecule’s ground state) is highly idealised.

In order to support this claim, it is important to first define what is meant by ‘isolation’. The term is understood in science in various ways; there is thermodynamic isolation, material isolation, radiative isolation, etc. Given how science understands ‘isolation’, a molecule is understood here as a system in isolation when the following requirements are met:

- The molecule is far removed from any other system and thus doesn’t interact with other molecular entities (i.e. with other atoms, molecules, ions, etc.); and,
- The total energy of the molecule is conserved.^14^

This is a highly idealised representation of a molecule for three reasons. First, it is very difficult to empirically examine a molecule’s properties in an environment in which the molecule doesn’t interact with other molecular entities. In practice, when it comes to the empirical support of chemical properties, the experiments are performed on chemical substances and not on a single molecular entity. Given this, it is common in chemical practice to assign chemical properties to a single molecule by measuring properties of samples of matter. The experiments are performed in such conditions that it is safe to assume that the behaviour of each molecule of an ensemble, closely resembles the behaviour of a single molecule when in isolation.^15^

This is merely an epistemic difficulty which, with the improvement of technological means, could possibly be overcome.^16^ There is, however, a second reason why the description of an isolated molecule is highly idealised. The empirical identification of a molecule’s stability (or of any other property) necessitates the interaction of that molecule with an experimental device, through the emission of light or the imposition of a field to the system. Therefore, the properties of an examined system are necessarily those that the system exhibits when it is in a state of non-conservation of energy. This cannot be overcome; in principle any act of measurement will probe the examined system in such a way that it will cease to be in isolation.

Thirdly, there is one additional feature that renders the chemical and quantum mechanical descriptions of an isolated molecule an idealisation; namely the way in which they represent it in time. To the extent that the stability of an isolated molecule is taken to correspond to its ground state, the stability of an isolated molecule is defined independent of time. This is because the ground state of a molecule is a stationary state; namely a state that ‘does not evolve with time’ (IUPAC 2014, p. 1443). Taking stability to be a time-independent property is an idealisation because in practice the stability of a molecule is always relevant to the particular time range of an experiment. In fact, there is empirical evidence of particular molecules which change from one structure into another in the course of time, suggesting that a molecule may not remain in one stable state indefinitely. For example (Meléndez-Martínez et al. 2014) examine the time-dependence of the different isomeric structures of carotenoids. So, given that in non-isolation, stability is a time-dependent property, it may be assumed that stability in isolation is a time-dependent property as well. On the other hand, one can assume that the stability of an isolated molecule is time-independent, in accordance to how it is described in quantum mechanics through the stationary states. While both assumptions are consistent with empirical evidence, the above analysis suggests the following. When describing an isolated molecule, chemistry’s and quantum mechanics’ understanding of stability as a time-independent property is an idealisation; namely something that is assumed, rather than empirically identified.

In sum, the stability of a molecule is empirically verified always in relation to particular factors, including the thermodynamics conditions, the environment, and the time-range of an experiment. Given this, the stability of an isolated molecule refers to a state of the molecule which can never be empirically identified and whose existence is assumed, rather than empirically verified. This idealisation is made both in chemistry and in quantum mechanics, whenever the two theories describe a stable isolated molecule. The next section argues that a similar idealised understanding is assumed with respect to the structure of an isolated molecule.

### Structure

Molecular structure is a central property of molecules that figures in the explanation of their chemical behaviour. The particular structure of a molecule can explain (to a certain degree) the stability and reactivity of that molecule. Also, the structure of a molecule that participates in a reaction may partially explain the process through which it transforms into particular products (i.e. the reaction mechanism), as well as the sort of products that are produced during the reaction. Moreover, physical properties of matter may be explained in terms of the structure of the molecules that compose it.

While molecular structure is employed by IUPAC for the definition of other chemical terms (such as molecular modelling and the Lewis formula), the term itself is not defined in the Gold Book. In light of this, this paper proposes a definition which is based on how the term is broadly understood in chemistry. Moreover, similarly to the examination of stability, this paper investigates how a molecule’s structure is understood and empirically identified for two cases, namely:

- a single molecule which is part of an ensemble; and,
- a single molecule in isolation.

Two concepts are often employed when referring to molecular structure; ‘shape’ and ‘conformation’. The ‘structure of a molecule’ is often employed interchangeably with the ‘shape of a molecule’ or ‘molecular shape’ (Ramsey 1997; Woolley 1976, p. 28). Moreover, depending on the type of molecules that are examined, molecular structure is often referred to in terms of the conformations that a specific molecule can take. Furthermore, the specific structure of a particular molecule is described by specifying the spatial arrangement of the atoms within that molecule.

Given the above, the structure of a molecule is defined here as the spatial arrangement of the atoms that constitute it. The factors that determine this spatial arrangement are contingent not only on the identity and interactions between the atoms that comprise the molecule, but also on the context in which the molecule as a whole is considered. This becomes evident when examining the structure of a molecule that is considered as part of an ensemble. In this case, the spatial arrangement of a molecule’s atoms is determined by (i) the interactions between the molecule’s own atoms (i.e. the intramolecular interactions), and (ii) the interactions between the molecule’s atoms and one or more distinct molecular entities (i.e. the intermolecular interactions).^17^

Molecular structure is a collective term in the sense that it doesn’t refer to one particular and empirically measurable chemical property, but rather to a collection of empirically measurable chemical (but also quantum mechanical) properties of the molecule. This seems to be in accordance with intuition. For example, when describing the structure of a box, one specifies its angles, the length of its sides, the distance between its surfaces, etc. There is no one measurable property that fully identifies and describes the structure of a box. The same applies to a molecule’s structure. There is a set of properties that need to be specified in order to provide a satisfactory description of molecular structure.

Given this, the properties that specify a molecule’s structure when that molecule is part of an ensemble, can be categorised into two sets. The first set includes the information that specifies the intramolecular interactions. This includes reference to:

- the types of intramolecular interactions, both bonding and nonbonding;
- the properties that are assigned to the intramolecular interactions;
- certain properties that are based on the quantum mechanical description of molecules;
- the pictorial representation of molecules; and,
- the properties that specify the structural differences between molecules.

Moreover, the structure of a molecule can be significantly affected by its intermolecular interactions. For example, the helical structure of DNA is determined by the intermolecular interactions (mainly hydrogen bonds) between the nucleic acids of the two strands that make up the DNA. These two strands are distinct molecules, and the reason why these two strands curl up into the overall helical structure of DNA (and why therefore they acquire their particular structure) is due to the intermolecular interactions between them. Another example is the structure of a water molecule (H_2_O). Two water molecules in a water dimer [i.e. (H_2_O)_2_] do not have the same structure and each molecule’s structure in the water dimer is also different from the structure of a single water molecule (whether in gas-phase, liquid-phase or solid-phase water) (Klopper et al. 2000).^18^ In light of this, there is a second set of properties which are invoked for the specification of a molecule’s structure. This set includes reference to:

- the types of interactions between the molecule (or its constituting atoms) and one or more distinct molecular entities; and,
- the properties that are assigned to those interactions.

This set is dependent on the identity of the molecule under examination, but also on the identity of the surrounding molecular entities.

Depending on the explanatory, predictive, and heuristic needs of chemistry as well as on the particular type of molecule that is under examination, intermolecular interactions may be disregarded in the description of a molecule’s structure. For example, a molecule with aromatic character exhibits unusual stability due to its structure, regardless of the particular environment in which it is considered (IUPAC 2014, p. 109). Therefore, its structure and stability are usually explained independently of the environment and conditions in which the molecule may be found. On the other hand, there are also cases where intermolecular interactions play an important role in explaining molecular structure, and thus in the behaviour of molecules. For example, the ‘abnormal physical properties’ of matter composed of NH_3_, H_2_O or HF is explained with reference to the intermolecular interactions that take place between such molecules (namely in terms of hydrogen bonding) (Needham 2013, p. 52).

In light of the above, it is clear that when a molecule is considered as part of an ensemble then its structure is a relational property, in the sense that a molecule’s structure is partially determined by the relations it develops with other molecular entities.

Concerning an isolated molecule, it becomes evident that, in virtue of being isolated, intermolecular interactions do not play any role in the determination of the molecule’s structure. However, it is not entirely accurate to infer from this that what determines an isolated molecule’s structure are only its intramolecular interactions. Just like in the case of stability, molecular structure is empirically supported in the context of chemical species, and not of an isolated molecule. The terms employed for the description of molecular structure are defined with reference to chemical species and empirically supported by experiments done on ensembles of molecular entities. Moreover, the structure of a single molecule is partially determined by the thermodynamic conditions in which the molecule is considered, as well as by the environment in which it is found.^19^ Therefore, the structure of an isolated molecule refers to an idealised state of the molecule which can never be empirically identified and whose existence is assumed rather than empirically verified.

Consider, for example, the empirical identification of chirality; namely of the ‘geometric property’ of a molecule ‘of being non-superposable on its mirror image’ (IUPAC 2014, p. 269).^20^ A chiral molecule is experimentally identified through the measurement of the optical activity of the sample in which that molecule is contained (IUPAC 2014, p. 1030). Any assembly of chiral molecules that is either enantiomerically pure (i.e. it contains only right or only left-handed enantiomers) or that contains an unequal amount of a pair of enantiomers (i.e. it contains, say, more left- than right- handed enantiomers) is optically active. The measurement of optical activity is performed on a sample of material, i.e. on matter composed of an ensemble of (either chemically distinct or identical) molecular entities. While chemists infer from such experiments that the observed sample contains molecules with specific chiral structure, the observed optical activity is that of the entire sample, and not of a single chiral molecule.

It should be noted that there are advances on single-molecule chemistry concerning the measurement of the polarisation of light produced by one single molecule. This is directly relevant to the empirical examination of the chirality of a single molecule (Chuntonov and Haran 2013). However, even in single-molecule chemistry, the single molecule whose properties are measured, is considered in a system where it interacts with other molecules. It is not the case that the single molecule is far removed from other molecular entities. Therefore, both the presence of other molecules, as well as the environmental and thermodynamic conditions in which a molecule is considered, are still relevant to the determination of its structure.

An additional idealisation concerning the structure of a molecule becomes apparent from how structure is pictorially represented. In chemistry, the structure of a molecule is represented statically, as if the molecule and its constituting atoms are not continuously moving and rotating in space. However, the pictorial representation of a molecule’s structure only highlights the average shape of a molecule, and does not refer to a static and unchanging structure. This is important because, even if one considers an isolated molecule, it is still the case that the particles that comprise an isolated molecule interact with each other and move in space. This is due to the existence of intramolecular interactions which persist even when the total energy of the system is constant (since the angular momentum of its comprising entities is non-zero). Therefore, the structure of a molecule is dynamic in the sense that a molecule (even in isolation) can possess different conformations in time as a result of the continuous interactions of the entities (namely electrons and nuclei) that comprise the molecule (Longuet-Higgins 1963, p. 446).

There is one additional factor that plays a central role in the understanding of structure; namely time. The empirical identification of a molecule’s structure is only possible if the examined molecule is stable for a particular range of time. If a molecule is not stable under particular conditions and within a particular environment, then it is not possible to empirically identify its structure. Given that the empirical identification of the stability of a molecule is relevant to the time-range of an experiment, it follows that the empirical identification of a molecule’s structure is also relevant to that particular time-range. This is further supported by the fact that there are types of molecules whose structure changes in the course of time (Meléndez-Martínez et al. 2014). All the above is not taken into account in quantum mechanics which describes the properties of a molecule by specifying its stationary (i.e. time-independent) states.

All the above illustrate the idealised framework in which chemistry and quantum mechanics each describe the structure of an isolated molecule.

## Philosophical implications

The previous section presented the factors that determine a molecule’s stability and structure. A molecule’s stability and structure are by necessity empirically identified in relation to those factors. Hence, when one describes a molecule in isolation, it is just assumed that it is stable and has structure, because this description (whether chemical or quantum mechanical) disregards the factors that partially determine the stability and structure of an empirically identifiable molecule. Whether this assumption is justified (namely whether an isolated molecule is stable and has structure) is not examined here. The paper only points out that, given how science empirically identifies these two properties, an isolated molecule with stability and structure is an idealisation.

If one takes into account the philosophical literature on idealisations, then numerous questions can be raised about the assumption that an isolated molecule is stable and has structure. First, there are various ways in which one could understand this idealisation. For example, one could define this as a deliberate simplification/distortion of the description of a molecule which disregards some of the factors that determine the molecule’s properties, in order to achieve one or more goals (i.e. tractability, solubility, simplicity, generality, identification of core causal factors, etc.) (in line with McMullin 1985; Weisberg 2007). On the other hand, one could say that chemistry and quantum mechanics each refer to a new system (i.e. an isolated and time-independent molecule) some of whose properties approximate some of those of the target system (i.e. a non-isolated and time-dependent molecule) (in line with Norton 2012). Contrary to both views, it could be argued that there is no interesting difference between these two interpretations of the idealisation; both interpretations capture the same understanding of idealisation regardless of whether we formulate it in terms of descriptions or in terms of the system to be described. However, there are philosophers who would possibly disagree with this. For example, Norton argues that his proposed understanding of idealisation introduces ‘reference to a novel system’ and thus carries ‘a novel semantic import’ (2012, pp. 208–209).

Another question is whether the assumption that is presented here is an example of a particular type of idealisation. For example, one could say that this is an example of Galilean idealisation as the latter is defined by Weisberg:

Galilean idealization is the practice of introducing distortions into theories with the goal of simplifying theories in order to make them computationally tractable. One starts with some idea of what a nonidealized theory would look like. Then one mentally and mathematically creates a simplified model of the target. (2007, p. 640)

Alternatively, one could say that this is an example of Aristotelian idealisation, as defined by Frigg and Hartmann:

Aristotelian idealization amounts to ‘stripping away’, in our imagination, all properties from a concrete object that we believe are not relevant to the problem at hand. This allows us to focus on a limited set of properties in isolation. (2012, Section 1.1)

While it is possible to argue for different types of idealisations, there are particular types that do not apply to the idealisation presented here. For example, the idealisation is not a case of an infinite idealisation where the latter is defined as ‘distortions or misrepresentations (…) in which some parameter takes on an infinite or infinitesimal value’ (Shech 2018, p. 1). In fact, it is not a case of a mathematical idealisation, in the sense that some mathematical value that is involved in the description of the system, is set to infinity or zero. Consider, for example, McMullin’s definition of mathematical idealisation:

Mathematical idealisation is a matter of imposing a mathematical formalism on a physical situation, in the hope that the essentials of that situation will lend themselves to mathematical representation. (1985, p. 254)

That the particular assumption is not a case of an infinite or mathematical idealisation is supported as follows. Indeed, quantum mechanics imposes a mathematical formalism on molecules via the Schrödinger equation. The equation assumes that an isolated molecule is stable and has structure by setting the values of some parameters in the Schrödinger equation. However, the same does not apply to chemistry’s description of a molecule. This is because chemistry is not primarily a mathematical description; its description of the relevant phenomena includes non-mathematical vocabulary as well. While it employs mathematical formalisms (from thermodynamics, quantum mechanics, etc.), it also employs a formalism that is non-mathematical. For example, chemistry’s definition and understanding of stability involves reference to chemical reactions which in turn are described with reference to the elements of the periodic table.

This issue also raises the question of why this idealisation is made in chemistry and in quantum mechanics. Since this idealisation is not only made in the mathematical formalism of quantum mechanics, it is inadequate to claim that its function is the mathematical tractability of the system. Perhaps its function is simplicity in general, in the sense that one includes in the descriptions as little as possible (Weisberg 2007, p. 650). Alternatively, its function may also be explanatory because the descriptions employ this idealisation in order to ‘include only the core causal factors which give rise to a phenomenon’ (Weisberg 2007, p. 642). While this section does not examine and specify in detail what the function of this idealisation is, it is evident that this idealisation serves more than one function in the relevant descriptions. First, this idealisation accommodates the mathematical tractability of the quantum mechanical description of a molecule. This is because, if quantum mechanics took into account time and the different conditions in which the molecule can be considered, then the relevant mathematical equation would be very difficult, if not impossible, to solve. Moreover, this idealisation has an explanatory function in the following sense. It is much easier for chemists to explain and understand the properties of a molecule, if they consider it in isolation and independent of time. By considering a molecule in isolation, chemists identify particular properties of the molecule and explain how these properties account for macroscopic properties of the matter in which the molecule is part of. These properties play a large role in understanding why the molecule behaves the way it does, even if these properties are not the only factors which determine its behaviour (see also Weisberg 2007, p. 650).

One more issue that is revealed in light of the literature on idealisations, concerns the nature of stability and structure. If stability and structure are only empirically identifiable when external factors partially determine them, then it is a matter of philosophical debate whether stability and structure are intrinsic or relational properties of a molecule. Put differently, can an isolated molecule be stable and have structure? Is the assumption of an isolated stable molecule with structure a ‘legitimate idealisation’ or an ‘outright falsehood’ (Ladyman 2008, p. 360)? This is a particularly interesting question, if one considers examples of idealisations in science which are considered as false or impossible:

For example, a perfectly reversible (or maximally efficient) Carnot engine is impossible to build in practice, and yet is considered a respectable part of the subject matter of thermodynamics. On the other hand, a perpetual-motion machine of the second kind, the sole effect of which is the complete conversion of heat into work, is regarded as fundamentally impossible. What is the difference between an impossibility that can be considered possible in ideal circumstances and an impossibility that remains so no matter how idealised the scenario we envisage? (Ladyman 2008, pp. 360–361)

Lastly, there is a general consensus in the philosophy of chemistry literature that the manner in which quantum mechanics describes an isolated molecule’s structure is problematic when it comes to specifying the relation between chemistry and quantum mechanics.^21^ Specifically, the inability of quantum mechanics to recover a molecule’s structure is posed as a challenge to the strict Nagelian reduction of chemistry to quantum mechanics, as well as to reductive and non-reductive physicalism (see for example Hendry 2010, p. 212; Woolley 1998).^22^ Identifying that the chemical and quantum mechanical descriptions of an isolated molecule involve the use of idealisations, can accommodate one’s understanding of quantum mechanics’ description of molecular structure, as well as of the relation between chemistry and quantum mechanics. These issues go beyond the scope of this paper. Nevertheless, the manner in which philosophy investigates the nature, type, and function of idealisations indicates that the identification of idealisations in chemistry and in quantum mechanics can provide novel insight into the relation between the two theories.

## Conclusion

When chemistry and quantum mechanics each describe an isolated molecule, they assume that the molecule is stable and has structure. This paper showed that these assumptions are an idealisation. This is because stability and structure are relational properties when referring to a molecule that is part of an ensemble. In fact, given that the empirical identification of a molecule’s stability and structure requires taking into account the environment, the thermodynamic conditions and the time-range of an experiment, it follows that chemistry and quantum mechanics assume, rather than empirically verify, that an isolated molecule is stable and has structure. The paper examined these assumptions in the context of the literature on idealisations and outlined the philosophical questions that are informed by this idealisation, including the question about the relation between chemistry and quantum mechanics.

## Footnotes

1 McMullin, as well as Frigg and Hartmann, formulate a definition which allows both understandings of idealisations; namely as a feature of theories and as a feature applied to systems or phenomena in the world.

2 The former is supported by Norton (2012). An example of the latter is (Wayne 2012, p. 343). A similar issue arises with respect to the distinction between idealisations and abstractions (Cartwright 1989; Ladyman 2008, pp. 362–363).

3 Specifically, ‘some scale models would also qualify as idealized models and it is not clear where exactly to draw the line between idealized and analogue models’ (Frigg and Hartmann 2012, Section 1.1). This question requires a detailed analysis of models and representations in science and is not examined here (for example Ladyman 2008, pp. 360–364).

4 The particular example may be alternatively identified as an abstraction or approximation. This is not examined here.

5 This paper leaves it open whether there are other features in chemistry or quantum mechanics which have not been considered as idealisations in the philosophical literature and which could possibly be construed as such.

6 A central reference of this paper is the Gold Book (IUPAC 2014) which is provided by the International Union of Pure and Applied Chemistry (IUPAC). IUPAC is one of the leading authorities regarding chemical nomenclature and the definition of chemical terms. The glossary of the Gold Book is more or less accepted by the entire chemical community; the terms and their respective definitions have been rigorously reviewed and published in chemistry journals.

7 Llored (2012) also points out factors that are involved in the empirical identification and description of molecules. However, contrary to the present paper, those factors are taken by Llored to include the relevant tools, methods, instruments and modes of access. Given this, as well as that he does not discuss this issue with respect to idealisations, this paper does not further consider his account.

8 What is meant by ‘isolation’ is explained later on in this paper.

9 A chemical species is ‘(a)n ensemble of chemically identical molecular entities that can explore the same set of molecular energy levels on the time scale of the experiment’ (IUPAC 2014, p. 264).

10 The term ‘conformation’ standardly applies to molecules which exhibit different stereoisomeric structures. However, it is also understood more broadly as referring to the different possible spatial arrangements of the atoms that comprise a molecular entity. The latter understanding of conformation is assumed here.

11 For example (Marvel 2009) examines stability with respect to various thermodynamic factors, including temperature.

12 Ney’s definition of relational properties provides a rather general understanding of relational properties because it does not specify what renders an object in relation to something else. This understanding of relational properties suffices in order to support the claim that the stability of a molecule that is part of an ensemble is not a property that the molecule has in and of itself.

13 Intrinsic properties are ‘properties objects have just in virtue of how they are in themselves, not how they are in relation to other things’ (Ney 2014, p. 285).

14 To my knowledge, there is no bibliographical reference in chemistry or quantum chemistry which explicitly specifies what renders a molecule in isolation (for example IUPAC 2014). However, based on how chemists and quantum chemists describe an isolated molecule and on how physics defines isolation, I take this to be a correct definition of an isolated molecule.

15 One could justify this assumption if, for example, the chemical substance is examined in very low pressure.

16 In fact, there has been extensive development of techniques and experiments in chemistry which aim at measuring the value of properties of single molecules, instead of inferring the properties of a single molecule by measuring the properties of an ensemble of molecules. This research is often referred to as ‘single-molecule chemistry’ (Bai et al. 1999; Walter 2008).

17 This justifies why this paper distinguishes between the structure of a single molecule considered as part of an ensemble, and the structure of a single molecule in isolation.

18 Needham raises a similar point regarding the properties of a molecule: ‘A molecule in the liquid isn't like a water molecule in isolation (in the gas phase at very low pressure), but will be subject to all the stresses imposed by the ambient medium’ (2017, p. 135).

19 For example, depending the temperature of the system, the molecule may exhibit different structures (Xiao et al. 2016).

20 A molecule has the property of being chiral when its mirror molecule is not identical to itself. The term ‘enantiomer’ refers to each molecule of any pair of chiral molecules. The two enantiomers of a pair of chiral molecules are often referred to as the right- and left-handed enantiomers.

21 A concise presentation of this is provided by Hettema (2017, pp. 49–68).

22 Strict Nagelian reduction is defined here as the position which at least requires the (in principle) derivation or deduction of chemistry from quantum mechanics (this is in line with Needham 2010, p. 164; Scerri 1994, p. 160). While there are different accounts of physicalism in the philosophy of science literature, physicalism (whether reductive or non-reductive) is broadly understood here as the ‘ontological position according to which the physical facts determine all the facts’ (Hendry 1999, p. 118).

## References

[CR1] Bai C, Wang C, Sunney Xie X, Wolynes PG (1999). Single molecule physics and chemistry. Proc. Natl. Acad. Sci. U.S.A..

[CR2] Batterman RW (2011). Emergence, singularities, and symmetry breaking. Found. Phys..

[CR3] Cartwright N (1983). How the Laws of Physics Lie.

[CR4] Cartwright N (1989). Nature’s Capacities and Their Measurement.

[CR5] Chuntonov L, Haran G (2013). Optical activity in single-molecule surface-enhanced Raman scattering: role of symmetry. MRS Bull..

[CR6] Frigg, R., Hartmann, S.: Models in Science, Stanford Encyclopedia of Philosophy. https://plato.stanford.edu/entries/models-science/ (2012). Accessed 15 July 2018

[CR8] Hendry RF (1999). Molecular models and the question of physicalism. HYLE.

[CR9] Hendry RF, Macdonald C, Macdonald G (2010). Emergence vs. reduction in chemistry. Emergence in Mind.

[CR10] Hettema H (2017). The Union of Chemistry and Physics-Linkages, Reduction, Theory Nets and Ontology.

[CR11] IUPAC: Compendium of Chemical Terminology: Gold Book, Version 2.3.3. http://goldbook.iupac.org/pdf/goldbook.pdf (2014). Accessed 3 May 2018

[CR12] Klopper W, van Duijneveldt-van de Rijdt JGCM, van Duijneveldt FB (2000). Computational determination of equilibrium geometry and dissociation energy of the water dimer. Phys. Chem. Chem. Phys..

[CR13] Ladyman J, Psillos S, Curd M (2008). Idealization. The Routledge Companion to Philosophy of Science.

[CR14] Llored JP (2012). Emergence and quantum chemistry. Found. Chem..

[CR15] Longuet-Higgins HC (1963). The symmetry groups of non-rigid molecules. Mol. Phys..

[CR16] Marvel CS (2009). Thermally stable polymers. Pure Appl. Chem..

[CR17] McMullin E (1985). Galilean idealization. Stud. Hist. Philos. Sci..

[CR18] Meléndez-Martínez AJ, Paulino M, Stinco CM, Mapelli-Brahm P, Wang XD (2014). Study of the time-course of cis/trans (Z/E) isomerization of lycopene, phytoene, and phytofluene from tomato. J. Agric. Food Chem..

[CR19] Needham P (2010). Nagel’s analysis of reduction: comments in defence as well as critique. Stud. Hist. Philos. Mod. Phys..

[CR20] Needham P (2013). Hydrogen bonding: homing in on a tricky chemical concept. Stud. Hist. Philos. Sci..

[CR21] Needham P (2017). Macroscopic Metaphysics: Middle-Sized Objects and Longish Processes.

[CR22] Ney A (2014). Metaphysics: An Introduction.

[CR23] Norton JD (2012). Approximation and idealization: why the difference matters. Philos. Sci..

[CR24] Ramsey JL (1997). Molecular shape, reduction, explanation and approximate concepts. Synthese.

[CR25] Scerri, E.: Has chemistry been at least approximately reduced to quantum mechanics? In: PSA: Proceedings of the Biennial Meeting of the Philosophy of Science Association 1994, pp. 160–170 (1994)

[CR26] Shech E (2018). Infinite idealizations in physics. Philos. Compass.

[CR27] Strevens M (2017). The structure of asymptotic idealization. Synthese.

[CR28] Walter N (2008). Single molecule detection, analysis, and manipulation. Encycl. Anal. Chem..

[CR29] Wayne A (2012). Emergence and singular limits. Synthese.

[CR30] Weisberg M (2007). Three kinds of idealization. J. Philos..

[CR31] Woolley RG (1976). Quantum theory and molecular structure. Adv. Phys..

[CR32] Woolley RG (1998). Is there a quantum definition of a molecule?. J. Math. Chem..

[CR33] Xiao C, Wei C, Guo LW, Shen J (2016). Effect of temperature and stress on molecular structure and carbon monoxide generation of lignite from Kailuan mining area. Int. J. Min. Sci. Technol..

